# Protective effect of regular physical activity against diabetes‐related lower extremity amputation

**DOI:** 10.1111/1753-0407.70011

**Published:** 2024-10-22

**Authors:** Jae Won Kim, Kyung‐Do Han, Jun Hyeok Kim, Yoon Jae Lee

**Affiliations:** ^1^ Department of Plastic and Reconstructive Surgery College of Medicine, The Catholic University of Korea Seoul Republic of Korea; ^2^ Department of Statistics and Actuarial Science Soongsil University Seoul Republic of Korea

**Keywords:** diabetes mellitus, diabetic foot ulcers, lower extremity amputation, physical activity

## Abstract

**Background:**

Foot ulcers are a major complication of diabetes mellitus that increase morbidity and mortality in patients with diabetes, affect their quality of life, and increase the overall social burden. A considerable number of patients with diabetic foot ulcers (DFUs) require amputations every year.

**Methods:**

This nation population–based study included 1 923 483 patients with diabetes who underwent regular health screening through the National Health Insurance Service during January 2009 and December 2012. We investigated the association between changes in physical activity (PA) status and the incidence of lower extremity amputation (LEA). Based on changes in PA status, participants were categorized into four groups: “remained inactive,” “remained active,” “active‐to‐inactive,” and “inactive‐to‐active.”

**Results:**

Regular PA is an independent factor associated with a decreased risk of LEA in patients with diabetes. During the follow‐up period, 0.23% (*n* = 4454) of the patients underwent LEA. Compared with the “remained inactive” group, the “remained active” group were at the lowest risk of LEA (adjusted hazard ratio 0.5888; 95% confidence interval 0.524–0.66). A protective effect of regular PA against LEA was observed in the “remaining active” group.

**Conclusions:**

Our findings suggest a protective role of PA against LEA in individuals with diabetes. This highlights the importance of recommending appropriate levels of PA for patients with diabetes. The study also showed a dose–response relationship, indicating that engaging in vigorous‐intensity PA was most beneficial, and higher amounts of PA may provide additional benefits.

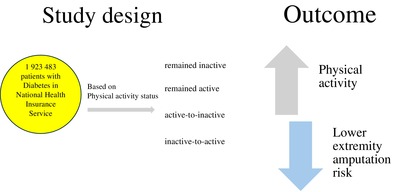

## Graphical abstract

- Regular physical activity has a protective effect lower extremity amputation in individuals with diabetes.
- There is evidence suggesting a proportional relationship between levels of physical activity and the reduction of amputation risk, with vigorous‐intensity activities demonstrating the most significant advantages.

## 1 INTRODUCTION

Between 15% and 25% of individuals with diabetes are likely to develop diabetic foot ulcers (DFUs) during their lifetime.^1^, ^2^, ^3^ Despite treatment, the risk of ulcer recurrence remains significant, with recurrence rates reported to be as high as 30%–40% within the first year.^4^ Approximately 85% of lower‐limb amputations result from non‐healing foot ulcers, and studies indicate a 15‐fold increase in lower‐limb amputations in diabetic patients compared with non‐diabetic patients.^1^, ^2^, ^5^ Despite medical interventions, the 5‐year survival rate of individuals with DFU is approximately 30%, and often exceeds to approximately 70% in cases with major amputation, emphasizing the need for effective prevention and management strategies.^5^

Peripheral neuropathy is a significant factor in the development of foot ulcers in diabetes, affecting approximately 66% of patients in the lower extremities.^6^, ^7^ This condition impairs sensory, motor, and autonomic functions due to hyperglycemia‐induced metabolic abnormalities.^2^, ^6^ Motor neuropathy, in particular, damages motor nerves, leading to foot deformities such as Charcot's foot, hammer toes, and muscle atrophy, which alter the foot's structure.^5^ The loss of protective sensation makes the feet more prone to injuries from ill‐fitting footwear, heat, and other harmful factors.^9^ Repeated injuries compromise skin integrity, creating pathways for microbial invasion and resulting in non‐healing wounds.^8^, ^9^ Additionally, autonomic neuropathy impairs sebaceous gland function, causing dry skin and fissures, which further increase the risk of wounds and infection.^9^

Peripheral vascular disease (PVD) is a major cause in around 50% of foot ulcer cases and is implicated in 70% of deaths related to type 2 diabetes.^10^, ^11^ Reduced blood flow to the extremities inhibits wound healing, increases the risk of infection, and can lead to chronic wounds or gangrene, often necessitating amputation.^11^ When PVD coexists with neuropathy, it becomes a primary risk factor for non‐traumatic amputations.^9^, ^10^, ^11^

Physical activity (PA), especially exercise therapy, has shown promising effects in regulating glycated hemoglobin (HbA1c) levels and improving the ankle‐brachial index (ABI).^12^ Considering that patients with diabetes frequently suffer from impaired foot sensation and atypical microvascular function in the foot skin, excessive pressure during PA can significantly increase the risk of developing DFUs.^12^, ^13^ Determining the appropriate level of PA presents a challenge for clinicians. Current guidelines generally advise that weight‐bearing exercises raise significant risks for these patients, particularly those with severe peripheral neuropathy, who should be encouraged to participate in non‐weight bearing activities instead.^12^, ^13^, ^14^

Recently, multiple prospective studies found that the individuals having developed ulcerations had lower PA levels than those without ulcerations. This can be explained by the fact that inactivity reduces stress tolerance of the skin, thereby increasing the risk of developing ulcers, and a sudden increase in pressure on the foot is a more important factor than the absolute amount of pressure.^15^, ^16^, ^17^

Therefore, it is crucial to investigate the appropriate level of PA or exercise for patients with DFUs. PA and exercise can improve nerve velocity conduction in the lower limbs, peripheral sensory function, and foot peak pressure distribution, which are all effective non‐pharmacological interventions to improve diabetic foot‐related outcomes.^18^, ^19^ Combined multidisciplinary treatments such as PA, diet routine, and foot care education are more effective in preventing foot complications in patients with diabetes.^18^

This study analyzed cohort data from the National Health Insurance Service (NHIS) in the Republic of Korea to assess whether regular exercise, as a modified behavioral factor, can reduce the incidence of limb amputation in patients with diabetes. Ultimately, we sought to determine the appropriate and regular levels of PA in patients at risk of diabetic complications.

## 2 METHODS

### 2.1 Database

This retrospective cohort study was conducted based on the data from the NHIS, a government‐run healthcare insurance system in South Korea that is conducted biennially and covers approximately 97% of Korean populations, excluding those receiving medical aid.^20^, ^21^ National health‐screening data provide inpatient and outpatient records of diagnostic codes, procedures, lifestyle questionnaires (drinking, smoking, and exercise), laboratory results, anthropometric measurements, and patient demographic information.^22^ Diagnostic codes were based on the International Classification of Diseases, 10th Revision.^23^ Thus, a nationwide population–based analysis would be useful for evaluating the association between risk factors and clinical outcomes.

The NHIS database was accessible to the researchers, and the study protocol was approved by the Institutional Review Board. Informed consent was not obtained for this study because the study included routinely collected data. The study was approved by the Institutional Review Board of Yeouido St. Mary's Hospital, Catholic Medical Center Office of Human Research Protection Program.(number: SC23ZISE0234).

### 2.2 Study population

**FIGURE 1:**
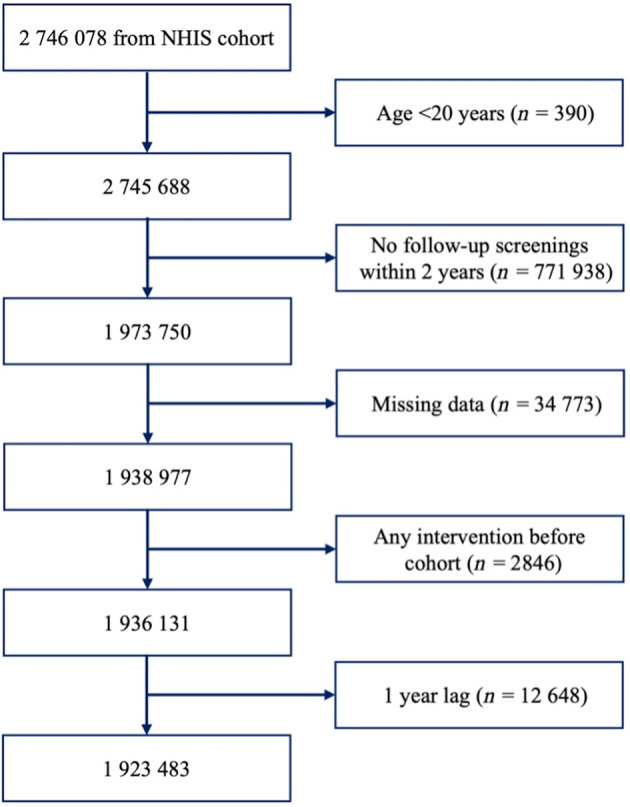
Flowchart illustrating the selection process for the study population. This flowchart outlines the selection process for the study population, utilizing data from the National Health Insurance Service (NHIS).

This study enrolled 2 746 078 patients diagnosed with type 2 diabetes who underwent health screening by the NHIS between January 2009 and December 2012. We selected 1 973 750 individuals aged >20 years who underwent follow‐up screening after 2 years. Patients with missing variables, any intervention such as traumatic lower‐extremity amputation before screening, and a 1‐year lag were excluded. In total, 1 923 483 individuals were included in this study (Figure 1).

### 2.3 Data collection

The primary endpoint of this study is the incidence of lower extremity amputation (LEA). We assessed the incidence of amputation based on the continuity and intensity of PA in patients with diabetes.

PA was measured using a modified Korean version of the International Physical Activity Questionnaire. Participants were questioned on the number of days they engaged in PA and at which intensity level in the week prior to examination. PA was classified into three categories based on intensity: vigorous, moderate, and walking. Vigorous activities included carrying heavy objects, running, aerobics, and fast biking. Moderate activities included carrying light items, biking at a normal speed, and playing tennis. Walking referred to usual‐pace walking for at least 10 min at a time.^24^ Regular PA was defined as vigorous activity for at least 20 min a day for three or more days per week or moderate‐intensity activity for at least 30 min a day for five or more days per week.^25^ PA was measured by determining the metabolic equivalent of tasks (METs). METs is widely used in medical research to quantitatively measure PA intensity.^26^ To yield the METs‐min/week measurements, we multiplied the metabolic equivalent values for each specific type of PA (for example, walking = 3, moderate activity = 5, vigorous activity = 8) by the number of minutes spent performing the activity in 1 week.^26^, ^27^

Other variables included age, sex, smoking status, alcohol consumption, body mass index (BMI), hypertension, chronic kidney disease, use of insulin and oral hypoglycemic agents, DM duration, serum glucose, white blood cell count, blood pressure, total cholesterol, lipoprotein, and triglyceride levels.

### 2.4 Statistical analysis

In this study, the statistical analysis was conducted using SAS version 9.4 (SAS Institute, Cary, NC, USA) and R version 3.2.3 (The R Foundation for Statistical Computing, Vienna, Austria, http://www.Rproject.org). Student's *t*‐tests and analysis of variance were used to examine continuous variables. The chi‐square test was used to investigate differences in categorical variables. While non‐parametric tests are recommended for variable that do not follow a normal distribution, in this study, variables such as triglycerides (TG) presented as a geometric means were log‐transformed, and a *t*‐test was performed on the log‐transformed TG. The mean difference test was conducted on the log‐transformed for other continuous variables. The PA score presented as a median; the *p*‐value was derived using the Brown–Mood median test. A Cox regression model was used to assess hazard ratios (HRs) and 95% confidence intervals (CIs) to investigate the independent relationship between PA and the risk of LEA. The cumulative incidence of LEA was plotted using Kaplan–Meier curves to explore other aspects of the data. Statistical significance was defined as a two‐sided *p*‐value of <0.05.

## 3 RESULTS

### 3.1 Demographic characteristics

Table 1 presents the demographics of the study population according to LEA. Among the 1 923 483 patients with diabetes, 0.23% (*n* = 4454) had undergone LEA. The amputated patient group generally comprised older patients and included more men and individuals who currently smoked, consumed alcohol heavily, did not exercise regularly, had a low income, had a lower BMI, were diagnosed with hypertension and/or chronic kidney disease (CKD), were treated with insulin or more than three oral antidiabetic drugs, had higher PA scores, and had higher serum glucose levels, WBC count, systolic blood pressure, triglycerides, and high‐density lipoprotein (HDL) levels than patients without LEA.

**TABLE 1:** Baseline characteristics of the study population of patients with diabetic foot based on National Health Insurance Service data.

	Total	Amputation		
		No	Yes	*p*
Number	1 923 483	1 919 029	4454	
Age (years)				<0.0001
20–40	129 617 (6.74)	129 518 (6.75)	99 (2.22)	
40–65	1 178 150 (61.25)	1 175 787 (61.27)	2363 (53.05)	
≥65	615 716 (32.01)	613 724 (31.98)	1992 (44.72)	
Sex				<0.0001
Male	1 183 393 (61.52)	1 179 893 (61.48)	3500 (78.58)	
Female	740 090 (38.48)	739 136 (38.52)	954 (21.42)	
Smoke				<0.0001
Non	1 061 175 (55.17)	1 059 176 (55.19)	1999 (44.88)	
Ex	394 209 (20.49)	393 165 (20.49)	1044 (23.44)	
Current	468 099 (24.34)	466 688 (24.32)	1411 (31.68)	
Drink				<0.0001
Non	1 096 895 (57.03)	1 094 220 (57.02)	2675 (60.06)	
Mild	655 208 (34.06)	653 909 (34.07)	1299 (29.16)	
Heavy	171 380 (8.91)	170 900 (8.91)	480 (10.78)	
Regular exercise	439 212 (22.83)	438 393 (22.84)	819 (18.39)	<0.0001
Income low (25%)	444 678 (23.12)	443 389 (23.1)	1289 (28.94)	<0.0001
BMI (kg/m^2^)				<0.0001
<18.5	28 721 (1.49)	28 565 (1.49)	156 (3.5)	
<23	503 505 (26.18)	501 962 (26.16)	1543 (34.64)	
<25	493 083 (25.63)	491 924 (25.63)	1159 (26.02)	
<30	766 067 (39.83)	764 660 (39.85)	1407 (31.59)	
≥30	132 107 (6.87)	131 918 (6.87)	189 (4.24)	
HP	1 095 082 (56.93)	1 091 927 (56.9)	3155 (70.84)	<0.0001
DYS	851 017 (44.24)	848 983 (44.24)	2034 (45.67)	0.0555
CKD	195 484 (10.16)	194 339 (10.13)	1145 (25.71)	<0.0001
Insulin	167 115 (8.69)	165 462 (8.62)	1653 (37.11)	<0.0001
≥3 oral hypoglycemic agent	319 697 (16.62)	318 358 (16.59)	1339 (30.06)	<0.0001
≥5 years since DM diagnosis	734 129 (38.17)	730 827 (38.08)	3302 (74.14)	<0.0001
PA score (median IQR)	435 (87–835)	435 (87–835)	348 (0–694)	<0.0001
Age (years)	58.36 ± 12.06	58.35 ± 12.06	62.53 ± 10.55	<0.0001
BMI (kg/m^2^)	24.92 ± 3.31	24.92 ± 3.31	24 ± 3.27	<0.0001
Glucose (mg/dL)	132.38 ± 45.43	132.29 ± 45.27	169.96 ± 83.6	<0.0001
WBC (×100/μL)	85.03 ± 8.59	85.03 ± 8.59	85.98 ± 8.61	<0.0001
SBP (mmHg)	127.93 ± 15.17	127.92 ± 15.16	131.02 ± 18.12	<0.0001
DBP (mmHg)	78.2 ± 9.93	78.2 ± 9.93	78.22 ± 10.87	0.8863
Total cholesterol (mg/dL)	189.63 ± 40.53	189.63 ± 40.51	188.95 ± 45.84	0.2669
HDL‐C (mg/dL)	51.04 ± 15.35	51.04 ± 15.35	49.33 ± 17.86	<0.0001
LDL‐C (mg/dL)	107.41 ± 37.64	107.42 ± 37.64	105.72 ± 40.93	0.0027
TG (mg/dL)^a^	136.19 (136.08–136.29)	136.16 (136.06–136.27)	146.47 (144.04–148.94)	<0.0001

*Note*: Values are expressed as the mean ± standard deviation, number (%), or median (interquartile range).

Abbreviations: BMI, body mass index; CI, confidence interval; CKD, chronic kidney disease; DBP, diastolic blood pressure; DM, diabetes mellitus; DYS, dyslipidemia; HDL‐C, high‐density lipoprotein cholesterol; HP, hypertension; IQR, interquartile range; LDL‐C, low‐density lipoprotein cholesterol; NHIS, National Health Insurance Service; PA, physical activity; SBP, systolic blood pressure; TG, triglycerides; WBC, white blood cell count.

^a^ Geometric mean (95% CI).

### 3.2 Risk of LEA in diabetic feet according to intensity, amount, and changes in PA

**FIGURE 2:**
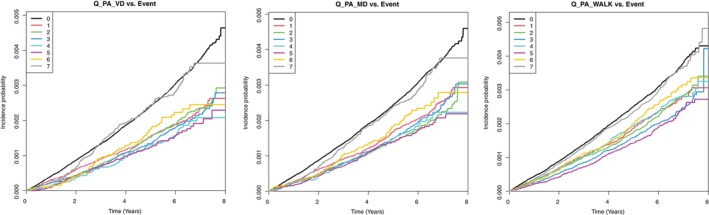
Kaplan–Meier curves depicting incidence probability of lower extremity amputation in diabetic population. Incidence probability of lower extremity amputation depending on (A) the duration of vigorous activity, (B) duration of moderate activity, and (C) walking duration.

The risk of LEA according to PA intensity, amount, and changes in patients with diabetes is presented in Table 2. Multivariate analysis was performed with or without adjustment for age, sex, smoking status, alcohol consumption, income status, BMI (kg/m^2^), hypertension, dyslipidemia, diabetes duration, insulin, and ≥3 oral hypoglycemic agents. Patients who engaged in PA 5 days per week demonstrated the lowest risk of LEA. Patients with vigorous activity showed the lowest risk (adjusted HR,^27^ 0.503; 95% CI,^1^ 0.409–0.62), followed by those with moderate activity (aHR, 0.552; 95% CI, 0.461–0.662) and walking (aHR, 0.618; 95% CI, 0.545–0.7). A Kaplan–Meier curve also showed that the incidence probability of LEA in diabetic feet was lowered by regular exercise of any type, including walking or moderate or vigorous activity. The reduction in the risk of LEA was most prominent in the vigorous activity group (Figure 2).

**TABLE 2:** Hazard ratio for risk of amputation in diabetic foot according to the duration and intensity of physical activity, regular exercise, physical activity score, and changes in physical activity levels at the initial and follow‐up screenings.

	Number	Event	Duration	IR per 1000	Model 1	*p*‐Value	Model 2	*p*‐Value	Model 3	*p*‐Value
Q_PA_VD										
0	1162 112	3115	6 186 811.71	0.50349	1 (Ref.)	<0.0001	1 (Ref.)	<0.0001	1 (Ref.)	<0.0001
1	223 785	391	1 234 509.87	0.31672	0.624 (0.562, 0.693)		0.675 (0.607, 0.751)		0.746 (0.67, 0.83)	
2	175 598	308	969 360.8	0.31774	0.626 (0.557, 0.703)		0.65 (0.577, 0.731)		0.714 (0.634, 0.804)	
3	143 816	236	794 437.28	0.29707	0.585 (0.512, 0.668)		0.58 (0.508, 0.662)		0.622 (0.545, 0.711)	
4	61 764	91	340 879.71	0.26696	0.526 (0.427, 0.648)		0.508 (0.412, 0.626)		0.543 (0.441, 0.669)	
5	63 124	91	345 491.82	0.26339	0.52 (0.422, 0.641)		0.485 (0.393, 0.597)		0.503 (0.409, 0.62)	
6	33 817	64	184 696.16	0.34652	0.685 (0.535, 0.877)		0.58 (0.453, 0.743)		0.585 (0.457, 0.75)	
7	59 467	158	321 367.86	0.49165	0.974 (0.83, 1.142)		0.765 (0.652, 0.898)		0.746 (0.636, 0.876)	
Q_PA_MD										
0	1 071 167	2900	5 719 308.05	0.50705	1 (Ref.)	<0.0001	1 (Ref.)	<0.0001	1 (Ref.)	<0.0001
1	209 407	394	1 149 380.93	0.34279	0.672 (0.605, 0.746)		0.752 (0.676, 0.836)		0.816 (0.734, 0.908)	
2	192 666	316	1 056 480.65	0.29911	0.587 (0.522, 0.659)		0.631 (0.561, 0.709)		0.681 (0.606, 0.766)	
3	171 654	304	941 341.19	0.32294	0.633 (0.563, 0.713)		0.647 (0.574, 0.728)		0.685 (0.608, 0.771)	
4	76 361	126	418 980.67	0.30073	0.59 (0.494, 0.706)		0.581 (0.486, 0.695)		0.611 (0.511, 0.731)	
5	79 707	123	433 446.26	0.28377	0.559 (0.467, 0.669)		0.536 (0.448, 0.642)		0.552 (0.461, 0.662)	
6	43 913	88	237 768.74	0.37011	0.729 (0.59, 0.901)		0.635 (0.513, 0.785)		0.633 (0.512, 0.783)	
7	78 608	203	420 848.72	0.48236	0.952 (0.826, 1.098)		0.775 (0.672, 0.894)		0.747 (0.647, 0.861)	
Q_PA_WALK										
0	587 574	1625	3 147 786.05	0.51624	1 (Ref.)	<0.0001	1 (Ref.)	<0.0001	1 (Ref.)	<0.0001
1	159 976	336	875 802.19	0.38365	0.74 (0.658, 0.832)		0.833 (0.74, 0.937)		0.88 (0.782, 0.991)	
2	209 095	426	1 135 717.59	0.37509	0.725 (0.652, 0.807)		0.8 (0.719, 0.891)		0.837 (0.752, 0.932)	
3	245 875	445	1 335 478.53	0.33321	0.644 (0.58, 0.716)		0.673 (0.606, 0.748)		0.694 (0.625, 0.771)	
4	137 362	292	745 063.94	0.39191	0.758 (0.669, 0.859)		0.763 (0.673, 0.864)		0.78 (0.689, 0.884)	
5	175 484	293	948 868.73	0.30879	0.598 (0.528, 0.677)		0.605 (0.534, 0.685)		0.618 (0.545, 0.7)	
6	118 752	270	644 014.17	0.41925	0.811 (0.713, 0.923)		0.763 (0.67, 0.868)		0.757 (0.665, 0.861)	
7	289 365	767	1 544 824.02	0.4965	0.964 (0.884, 1.05)		0.859 (0.788, 0.936)		0.835 (0.766, 0.91)	
Regular exercise										
No	1 484 271	3635	7 976 745.41	0.4557	1 (Ref.)	<0.0001	1 (Ref.)	<0.0001	1 (Ref.)	<0.0001
Yes	439 212	819	2 400 809.79	0.34113	0.746 (0.692, 0.805)		0.671 (0.621, 0.724)		0.673 (0.623, 0.726)	
PA score										
Q1	469 617	1400	2 500 165.36	0.55996	1 (Ref.)	<0.0001	1 (Ref.)	<0.0001	1 (Ref.)	<0.0001
Q2	504 081	1111	2 715 246.15	0.40917	0.73 (0.675, 0.79)		0.776 (0.717, 0.84)		0.797 (0.736, 0.863)	
Q3	468 185	1038	2 531 913.88	0.40997	0.731 (0.674, 0.792)		0.722 (0.666, 0.782)		0.738 (0.681, 0.8)	
Q4	481 600	905	2 630 229.81	0.34408	0.612 (0.563, 0.665)		0.569 (0.523, 0.619)		0.583 (0.536, 0.634)	
Regular exercise (pre/post)										
No/no	1 249 442	3031	6 701 550.05	0.45228	1 (Ref.)	<0.0001	1 (Ref.)	<0.0001	1 (Ref.)	<0.0001
No/yes	258 014	497	1 403 315.16	0.35416	0.781 (0.711, 0.859)		0.715 (0.65, 0.786)		0.713 (0.649, 0.784)	
Yes/no	234 829	604	1 275 195.36	0.47365	1.045 (0.957, 1.14)		0.926 (0.849, 1.011)		0.89 (0.815, 0.972)	
Yes/yes	181 198	322	997 494.63	0.32281	0.711 (0.633, 0.797)		0.593 (0.528, 0.665)		0.588 (0.524, 0.66)	

*Note*: Model 1: Non‐adjusted. Model 2: Age, sex. Model 3: Age, sex, smoke, drink, income, HP, DYS, BMI, insulin, oral hypoglycemic agents, DM duration. Q_PA_VD: The number of days per week on which vigorous exercise lasting 20 min or more was performed (0–7 days). Q_PA_MD: The number of days per week on which moderate exercise lasting 30 min or more was performed (0–7 days). Q_PA_WALK: The number of days per week on which walking exercise lasting 30 min or more was performed (0–7 days).

Abbreviations: BMI, body mass index; DM, diabetes mellitus; DYS, dyslipidemia; HP, hypertension; IR, incidence rate; PA, physical activity.

**FIGURE 3:**
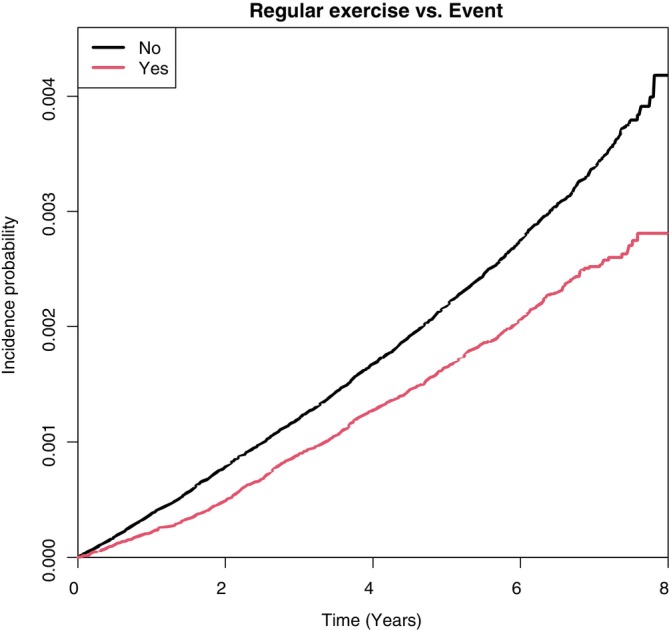
Kaplan–Meier curve showing cumulative reduction in lower‐extremity amputation. The Kaplan–Meier curve illustrates the cumulative reduction in the incidence of lower‐extremity amputation among diabetic individuals who engaged in regular exercise.

The risk of LEA decreased with regular exercise (HR, 0.673; 95% CI, 0.623–0.726) after adjusting for other variables like age, sex, smoking status, alcohol consumption, income status, BMI (kg/m^2^), hypertension, dyslipidemia, diabetes duration, insulin, and ≥3 oral hypoglycemic agents. A Kaplan–Meier curve also showed that the cumulative incidence of LEA in diabetic feet was lowered by regular exercise (Figure 3).

**FIGURE 4:**
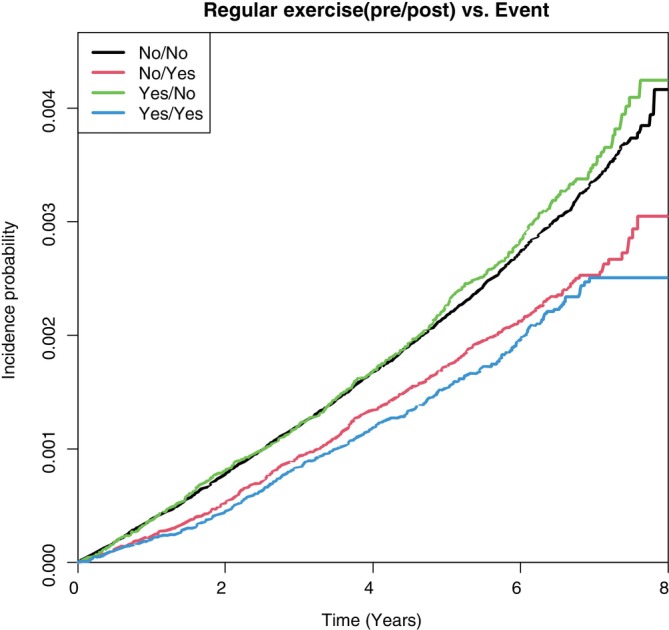
Kaplan–Meier curve depicting lower extremity amputation incidence in regard to different physical activity levels. Incidence of lower extremity amputation according to the changes in physical activity levels at the time of the initial and follow‐up screening in patients with diabetes.

The patients were divided into four groups based on the changes in their PA levels at the time of the initial and follow‐up health screenings. From the highest to lowest aHR, the groups were ranked as follows: “remained inactive,” “active‐to‐inactive” (0.89; 95% CI 0.815–0.972), “inactive‐to‐active” (0.713; 95% CI 0.649–0.784), and “remained active” (0.588; 95% CI 0.524–0.66) (*p* < 0.0001). The group of patients who engaged in regular PA at the follow‐up screening (groups “inactive‐to‐active” and “remained active”) exhibited a lower amputation rate (Figure 4).

## 4 DISCUSSION

Effectively managing and preventing amputation in DFUs is paramount not only for enhancing patient well‐being but also for reducing the economic burden associated with treatment costs. A key strategy is to meticulously control blood glucose levels.^14^ Numerous methods for avoiding DFUs have been proposed, including appropriate PA (structured PA or simple daily life movements), healthy food consumption, and medications.^15^ Implementing these comprehensive approaches not only promotes individual health but also serves as a cost‐effective means to reduce the occurrence and complications of DFUs.

In this study, we analyzed whether regular exercise, as a modifiable behavioral factor, is associated with the risk of LEA in Korean patients with diabetic foot disease. In this large‐scale population‐based cohort study involving more than 1.8 million patients with diabetes, we demonstrated a significant reduction in the incidence of LEA among individuals who engaged in regular PA for a minimum of 2 years. This association was evident regardless of sex and was particularly notable among patients aged 40 years and above. Furthermore, a higher intensity and greater frequency of PA were associated with further reduction in the risk of amputation.

Risk factors for LEA include male sex, smoking history, previous diabetic ulcers, lower BMI, diabetic neuropathy, peripheral artery disease (PAD), foot deformities, poor glycemic control, elevated glycated hemoglobin (HbA1c) levels, hypertension, renal complications, prolonged diabetes duration, and obesity.^28^, ^29^, ^30^

PA exerts protective effects against LEA.^31^ According to recent research, regular PA and exercise can improve the dynamic plantar pressure distribution, nerve conduction velocity, peripheral sensory function, and foot mobility, thereby promoting better involvement of the entire foot during walking. Exercise can also delay the typical progression of diabetic peripheral neuropathy, which is a significant risk factor for skin breakdown and amputation, and reduce the occurrence of skin damage and diabetic ulcers.^18^, ^32^ Some studies have reported that the PA group experienced a decrease in peak pressure on the plantar surface and improvement in muscle function.^33^ These findings suggest that exercise has a beneficial effect on reducing the complications of diabetic ulcers.^34^

In the past, it was believed that PA could increase the risk of foot ulcers in diabetic patients with impaired sensation in the feet.^33^ However, since 2008, new evidence has suggested that weight‐bearing activities do not increase the incidence of foot ulcers. This finding has prompted the call for a paradigm shift towards enhancing PA.^35^

Recent evidence underscores the effectiveness of patient education in improving lifestyle behaviors, including PA. Coppola et al. provides compelling data supporting the role of structured education in enhancing outcomes for diabetic foot patients.^13^ The study found that patients who participated in regular educational sessions, which included guidance on safe and appropriate activities, had better outcomes in terms of DFU compared to those who did not receive such education.^13^ By authorizing patients with knowledge and practical tools, we can help them adopt a more active lifestyle, ultimately reducing the risk of diabetic foot complications and enhancing their overall quality of life.

The American Diabetes Association recommends that all adults with type 2 diabetes reduce their sedentary time and that a combination of aerobic and resistance exercise training is necessary for optimal blood glucose management and outcomes.^35^ For most adults, this includes 150 min or more of moderate‐to‐vigorous activity weekly, and 2–3 sessions per week of resistance exercise.^35^

More recently, Streckman et al. showed that aerobic training of at least moderate intensity (3–6 times per week) for 12 weeks could improve glucose control and peripheral nerve conduction.^36^ Sensorimotor training has also been shown to contribute to balance control and target the signs and symptoms of peripheral neuropathy.^36^

Smoking is a significant factor leading to peripheral arterial and cardiovascular diseases, and is considered an important factor that increases the risk of LEA in patients with DFUs. Additionally, an inverse association between BMI and amputation risk was observed, suggesting that a lower BMI is associated with an increased likelihood of LEA. The paradoxical protective effect of a higher BMI may be due to better nutritional status and an increased ability to deal with severe infections.^30^

In our study, we gathered PA data from more than 1 900 000 individuals diagnosed with diabetes at two different time points separated by a 2‐year gap prior to the onset of LEA. In addition, we conducted an extensive survey covering almost the entire Korean population using Korea's comprehensive health insurance system. Regular PA was operationally defined as engaging in vigorous activity for a minimum of 20 min per day on at least 3 days a week or engaging in moderate‐intensity activity for a minimum of 30 min per day on at least 5 days a week. Our findings showed that maintaining regular PA regimen yielded a significant reduction in amputation rates.

## 5 CONCLUSIONS

Regular PA emerged as an independent factor associated with a notable reduction in the risk of LEA in patients with diabetes. This protective effect was observed regardless of the patient's sex. Notably, our study revealed a dose–response relationship, indicating that engaging in vigorous‐intensity PA was the most beneficial, and higher amounts of PA may provide additional benefits. Our findings provide strong quantitative evidence that PA enhances LEA prevention in patients with diabetes.

## AUTHOR CONTRIBUTIONS

*Conception or design*: Kyung‐Do Han and Yoon Jae Lee. *Acquisition, analysis, or interpretation of data*: Jae Won Kim, Jun Hyeok Kim, and Kyung‐Do Han. *Drafting the work or revising*: Yoon Jae Lee and Jae Won Kim. *Final approval of manuscript*: Yoon Jae Lee.

## CONFLICT OF INTEREST STATEMENT

The authors declare no conflicts of interest.

## Data Availability

The datasets used and/or analyzed in the current study are available from the corresponding author upon reasonable request.
